# The Impact of Diabetes on Brain Health in Childhood

**DOI:** 10.3390/biomedicines14030721

**Published:** 2026-03-20

**Authors:** László Barkai

**Affiliations:** 1 Department of Paediatrics and Adolescent Medicine, Faculty of Medicine, Pavol Jozef Šafarik University, Tr. SNP 1, 040 01 Kosice, Slovakia; laszlo.lajos.barkai@upjs.sk or barkai.laszlo@uni-obuda.hu; Tel.: +36-309384467; 2 Physiological Controls Research Center, University Research and Innovation Center, Obuda University, Bécsi út 96, 1034 Budapest, Hungary

**Keywords:** brain health, diabetes, children, adolescents

## Abstract

**Background/Objectives:** The global incidence of diabetes in childhood is increasing, raising concern about its long-term effects on the developing brain. Although paediatric diabetes research has traditionally focused on microvascular and macrovascular complications, accumulating evidence indicates that the brain is also a vulnerable target. **Methods:** This narrative review synthesizes current knowledge on the impact of diabetes on brain health in children and adolescents, with emphasis on epidemiology, neuroimaging and cognitive outcomes, underlying mechanisms, risk and protective factors, and clinical implications. **Results:** In type 1 diabetes (T1D), studies consistently demonstrate subtle but measurable alterations in brain structure, including reduced growth of total, grey, and white matter volumes, alongside functional and microstructural changes. These neurobiological differences are associated with mild deficits in cognition, particularly in attention, executive function, memory, and processing speed. While clinically significant impairment affects a minority, subclinical alterations are common and may accumulate over time. Key risk factors include chronic hyperglycaemia, glycaemic variability, severe hypoglycaemia, diabetic ketoacidosis, and younger age at onset, whereas good glycaemic stability, diabetes technologies, supportive psychosocial environments, and adequate sleep appear protective. Proposed mechanisms involve oxidative stress, neuroinflammation, disrupted insulin signalling, altered cerebral metabolism, and vulnerability of the immature brain during critical developmental windows. Type 2 diabetes (T2D), increasingly diagnosed in youth, is also associated with adverse brain outcomes. Emerging data link early-onset T2D to alterations in brain structure and connectivity, poorer cognitive performance, and increased mental health burden, mediated by hyperglycaemia, insulin resistance, inflammation, and psychosocial stressors. **Conclusions:** Overall, childhood diabetes—both T1D and T2D—is associated with meaningful effects on brain development and function. Longitudinal and interventional studies are needed to establish causality and determine whether optimizing glycaemic control and psychosocial support can mitigate neurocognitive risk. Recognizing brain health as a potential complication of paediatric diabetes has important implications for monitoring, prevention, and clinical care.

## 1. Introduction

The global incidence of diabetes in children and adolescents continues to rise, with both type 1 diabetes (T1D) and type 2 diabetes (T2D) imposing a lifelong metabolic burden from an early age. Advances in diabetes care have substantially improved survival, shifting clinical attention toward long-term complications. While microvascular and macrovascular sequelae are well characterised, the brain has only recently been recognised as a potential target of diabetes-related injury in childhood.

Brain development during childhood and adolescence involves dynamic maturation processes that may be particularly vulnerable to metabolic disturbances associated with diabetes. Increasing evidence from neuroimaging and neuropsychological studies indicates that childhood diabetes is associated with subtle but measurable alterations in brain structure, connectivity, and cognitive function, even in the absence of overt neurological disease.

In T1D, diabetes-related brain effects include subtle structural, functional, and cognitive differences, with clinically significant impairment affecting a minority but subclinical alterations appearing common and potentially progressive. In parallel, youth-onset T2D has been associated with adverse neurocognitive and mental health outcomes, likely reflecting the combined effects of metabolic and psychosocial factors.

This narrative review aims to summarise the impact of diabetes on brain health in children and adolescents. First, the paper addresses type 1 diabetes and the brain, including epidemiology, structural and functional neuroimaging findings, cognitive outcomes, and associated risk and protective factors. It then outlines the putative pathophysiological mechanisms underlying diabetes-related brain alterations. Subsequently, the emerging evidence on type 2 diabetes in youth and its neurodevelopmental and cognitive implications is examined. Finally, current gaps in knowledge, future research directions, and clinical implications are discussed. Scopus, PubMed, and Web of Science databases were searched for appropriate literature. The search was carried out using combinations of the terms: “type 1 diabetes”, “type 2 diabetes”, “children”, “adolescents”, “brain structure”, “brain functions”, “neurocognition”, “risk factors”, “diabetes onset”. The results are presented in a narrative manner.

## 2. Type 1 Diabetes and the Brain

### 2.1. Epidemiology of Brain Dysfunction in Childhood T1D

Childhood and adolescence are characterised by dynamic and region-specific brain maturation. Total brain volume increases rapidly in early childhood, followed by cortical thinning during later childhood and adolescence, reflecting synaptic pruning and greater neural efficiency. Gray matter volume peaks in late childhood and then declines, particularly in frontal regions, while white matter volume continues to increase into early adulthood due to ongoing myelination and strengthening of connectivity. Functional networks are progressively reorganised, supporting the development of executive functions, memory, and processing speed. Because these maturational processes rely on stable metabolic supply and insulin-sensitive signalling pathways, the developing brain may be particularly vulnerable to glycaemic disturbances associated with type 1 diabetes [1,2].

Type 1 diabetes is the most common form of diabetes in children and adolescents, and its incidence continues to rise [3]. Childhood and adolescence are characterised by rapid structural and functional brain maturation, including increases in white matter volume, synaptic pruning, and evolving functional connectivity. This critical developmental window may render the brain particularly susceptible to metabolic perturbations associated with diabetes [4].

Although the precise prevalence of brain-related complications in paediatric diabetes remains uncertain, meta-analyses and neuroimaging studies increasingly document cognitive and neuroanatomical differences between children with T1D and healthy controls. For example, meta-analytic data demonstrate significant cognitive deficits in children with T1D compared with peers [5].

Brain dysfunction in childhood-onset T1D is recognised as a complication, although reported frequency varies depending on how dysfunction is defined—ranging from subtle differences on neuropsychological testing to clinically meaningful impairment. Many paediatric studies do not report a single prevalence estimate but instead describe subclinical alterations in brain structure or function and modest reductions in cognitive test performance (e.g., processing speed, executive function, memory).

Earlier studies suggested that approximately 12.8% of children with T1D met criteria for clinically meaningful cognitive impairment, compared with ~5–6% of non-diabetic controls [6]. In a small MRI-based cohort study, central nervous system structural abnormalities were identified in 29% of children with T1D, including mesial temporal sclerosis in 16% [7]. In adults with childhood-onset T1D, approximately 28% exhibited clinically relevant cognitive impairment in mid-life, suggesting that effects may accumulate over decades [8].

Overall, brain involvement in childhood-onset T1D spans a spectrum from subtle, subclinical cognitive differences to clinically relevant impairment in approximately 10–30% of individuals, depending on age, disease duration, and assessment criteria. While clinically defined impairment affects a minority, subclinical alterations are observed in a substantially larger proportion of youth with T1D.

### 2.2. Structural Brain Changes

Structural MRI studies in children and adolescents with T1D consistently demonstrate subtle but regionally specific alterations in brain morphology. Beyond reduced total brain volume, several studies report lower gray matter and white matter volumes compared with controls, particularly in frontal and parietal regions [9,10,11,12]. In young children with early-onset T1D, longitudinal analyses have shown attenuated growth trajectories of both cortical gray matter and cerebral white matter over follow-up periods of approximately 18–24 months [13]. Larger longitudinal studies extending into early adolescence demonstrated persistent differences in total, gray, and white matter volumes, with higher lifetime HbA_1_c and cumulative exposure to hyperglycaemia associated with smaller brain volumes over time [12].

Region-specific alterations have also been reported. Reduced gray matter volumes in midbrain, thalamic regions, and cerebellar areas (including the cerebellar culmen) have been described in childhood-onset T1D [14,15]. Neuroanatomical correlates of dysglycaemia in young children further implicate posterior cortical and subcortical regions [10]. Earlier MRI-based cohort studies also reported structural central nervous system abnormalities, including mesial temporal sclerosis in a subset of children with early-onset T1D [7].

Diffusion-weighted imaging (DWI) studies indicate microstructural alterations in white matter tracts. Reduced fractional anisotropy and increased diffusivity have been observed in association with T1D, suggesting compromised white matter integrity [9,16]. In paediatric cohorts, white matter microstructural changes have been linked to hyperglycaemia and severe hypoglycaemia exposure [9], as well as to broader alterations in white matter structure in young children [11,17].

Functional MRI (fMRI) studies provide complementary evidence of altered neural activation patterns. Task-based fMRI using working memory paradigms (e.g., N-back tasks) demonstrated greater activation in frontal and parietal regions in children with T1D compared with controls, interpreted as compensatory recruitment [12]. Additional studies from the DirecNet consortium have linked dysglycaemia to neuroanatomical and functional differences in developing brain networks [10,12,16].

Collectively, structural MRI, diffusion imaging, and functional MRI studies converge in indicating that childhood-onset T1D is associated with subtle but regionally distributed alterations in brain structure and network organisation, likely reflecting deviations from typical neurodevelopmental trajectories rather than focal neuropathology.

### 2.3. Cognitive Deficits and Neuropsychological Outcomes

Children with diabetes exhibit subtle but measurable cognitive deficits across multiple domains: intelligence quotient (IQ), attention, memory, executive function, psychomotor speed. An Australian group followed a cohort of T1D children since the onset of disease for 12 years. As compared to controls, subjects were performing worse on working memory, attention, new learning and mental efficiency and had higher rates of mental health referrals and lower school completion. This long-term study has shown the emergence of cognitive deficits in children with T1D over time [18].

A meta-analysis of 19 studies (1355 children with T1D and 696 controls) found overall poorer cognitive performance, deficits in full-scale IQ, attention, and psychomotor speed [14]. Another older meta-analysis (24 studies) found small deficits in visuospatial ability, motor speed, writing, sustained attention, reading, and full IQ [19]. More recently, a cross-sectional study of children and adolescents with T1D found that poorer metabolic control (higher HbA_1_c), history of diabetic ketoacidosis (DKA), and younger age at onset were associated with lower IQ and increased impulsivity [20].

Developmental timing matters. Early-onset diabetes (in very young children) may overlap with periods of rapid brain growth (especially white matter) and thus may have greater impact. Some reviews conceptualise this via a developmental diathesis/stress model: early metabolic insults in a vulnerable period may lead to altered developmental trajectory [21]. A recent study in children aged ~11.5 yrs found that children with T1D performed worse on a working memory (N-back) task, and showed different brain activation patterns (greater modulation of activation) suggesting compensatory mechanisms [15].

In a longitudinal prospective study, the trajectories of cognitive performance were analysed in T1D children and non-diabetic siblings over time. Youth with type 1 diabetes performed worse than non-diabetic siblings on visual-spatial ability and memory tasks over time, and did not improve as much in processing speed. The results of this study suggest that on average differences in cognitive function between youth with T1D and non-diabetic relatives are maintained or increase during childhood and adolescence [22].

Although the cognitive differences are generally mild, they may have functional consequences in academic performance, adherence to diabetes regimens (which require cognitive and executive skills), and psychosocial outcomes. Screening for cognitive/attention difficulties may be appropriate in high-risk children (e.g., early onset, poor glycaemic control, history of DKA). Cognitive findings in paediatric diabetes are summarised in Table 1.

Overall, children and adolescents with type 1 diabetes exhibit mild but consistent differences across multiple cognitive domains, with effects that may persist or emerge over time and carry functional relevance for academic performance and diabetes self-management.

### 2.4. Risk and Protective Factors for Brain Damage

#### 2.4.1. Risk Factors

Historically, central nervous system abnormalities in childhood diabetes have been primarily attributed to recurrent **hypoglycaemia**, which is difficult to avoid during insulin therapy [24]. Subsequent longitudinal studies of participants in the Diabetes Control and Complications Trial (DCCT) suggested little, if any, long-term cognitive risk associated with hypoglycaemia occurring in older adolescents and young adults. However, a modest decline in psychomotor efficiency was observed in association with long-term metabolic control [25].

More recent studies have provided evidence that a history of three or more severe hypoglycaemic episodes is associated with alterations in white matter microstructural integrity in youths with type 1 diabetes (T1D), including reduced anisotropy and increased diffusivity in the superior parietal lobule, as well as increased diffusivity in the hippocampus [9]. Another study demonstrated that frequent and early exposure to severe hypoglycaemia during neurodevelopment negatively affects spatial long-term memory performance in children with T1D [26].

Neuroanatomical and cognitive alterations observed in participants recruited from The Diabetes Research in Children Network (DirecNet) Study Group were correlated with **hyperglycaemia** [10]. Neuropsychological evaluations of children with T1D and control subjects across DirecNet sites showed that the degree of hyperglycaemia was associated with impairments in executive function and, to a lesser extent, in IQ, learning, and memory [27]. In a prospective study, changes in brain volumes and cognitive performance persisted over time in children with early-onset T1D and were longitudinally associated with metrics of hyperglycaemia [12]. More recent longitudinal data further support the conclusion that long-term hyperglycaemia is an important risk factor for cognitive dysfunction in children and adolescents with T1D [22]. Additionally, a prospective study following brain volume over two years in youths with T1D and their non-diabetic siblings demonstrated that exposure to hyperglycaemia and severe hypoglycaemia may result in subtle deviations from typical brain developmental trajectories. Specifically, gray matter volume decline was associated with elevated HbA1c, whereas white matter volume decline was linked to hypoglycaemia [28].

Less is known about the effects of **extensive glucose fluctuations** on the developing brain in childhood diabetes. Preliminary evidence indicates that alterations in white matter structure are related to increased blood glucose variability, and that fluctuating glucose levels may be associated with corresponding fluctuations in brain volume in children with diabetes [11,13]. In a randomized, interventional six-month pilot study comparing conventional therapy with hybrid closed-loop systems, improvements in time in range (TIR) as a measure of glucose variability in adolescents with T1D were associated with quantifiable improvements in cognitive and neuroimaging outcomes over time, regardless of the treatment system used [29].

Several studies suggest that **younger age at diabetes onset** constitutes an additional risk factor [21], although some recent findings have questioned this association [30]. Early onset of T1D (before 7 years of age) has been linked to mild central brain atrophy and significant differences in intellectual performance in adulthood, suggesting that early disease onset may adversely affect neurodevelopment [31]. Structural MRI studies have further demonstrated that early-onset T1D has widespread effects on gray and white matter growth, even in children whose glycaemic control falls within current treatment guidelines [13]. A meta-analysis assessing cognitive function in children with T1D found mildly lower cognitive performance across most domains compared with control subjects, with the most pronounced and pervasive deficits observed in those with early-onset disease (<7 years), who exhibited moderately lower cognitive scores [32]. Furthermore, a large population-based cohort study reported an association between childhood-onset T1D and increased risk of depression, anxiety, and stress-related disorders, particularly among those with early disease onset. The study also suggested that shared familial factors may contribute to these elevated risks [33].

A **history of diabetic ketoacidosis (DKA)** is also considered a risk factor for brain dysfunction [23]. Presentation with DKA at the onset of childhood T1D has been associated with long-term detrimental effects on cognitive function, including lower IQ, impaired memory, and reduced executive function. These effects are particularly severe in young children (ages 3–5 years) and have been linked to structural brain changes and slower processing speed [34,35]. Recurrent episodes of DKA have also been associated with cognitive decline [12,36]. However, many of these studies were conducted over relatively short follow-up periods, ranging from several months to a few years, underscoring the need for long-term longitudinal studies spanning childhood to adulthood to better characterize the long-term sequelae of DKA on brain function. In addition, prior studies were often unable to exclude preexisting cognitive differences. To address this limitation, one study incorporated socioeconomic status (SES) into regression analyses and identified a significant interaction effect, whereby DKA status was associated with lower IQ among children with higher SES, while children with lower SES exhibited lower IQ regardless of DKA exposure [37].

**Sleep quality** may further influence diabetes-related cognitive dysfunction in children. Poor or disrupted sleep can worsen glycaemic control, increase glucose variability, and elevate stress hormone levels, all of which may adversely affect the developing brain. Nocturnal hypoglycaemia or hyperglycaemia may disrupt critical sleep stages necessary for memory consolidation and learning, leading to impairments in attention, processing speed, and executive function. Nevertheless, additional studies are required to clarify these associations in paediatric diabetes [33,38].

**Psychosocial factors** affecting parents and caregivers may also significantly influence diabetes-related cognitive outcomes in children through both emotional and physiological mechanisms. Parental stress, anxiety, depression, and diabetes-related burnout can interfere with consistent diabetes management, resulting in poorer glycaemic control and increased glucose variability. Elevated parental distress may contribute to family conflict, reduced adherence to treatment regimens, and disrupted sleep and emotional regulation in the child. Furthermore, children may internalize parental stress, which can negatively affect attention, learning, and executive functioning [23,38,39,40]. Family dynamics, parental involvement, and the quality of caregiver support influence adherence to treatment regimens, glycaemic control, and overall disease management [18,19]. Moreover, paediatric patient populations show correlations with the sociodemographic level of the family, including parental education, income, and caregiver well-being, which in turn affect the child’s clinical outcomes and psychological adjustment [19,20]. Psychological comorbidities such as anxiety, depression, and diabetes-related distress are prevalent in children and adolescents with T1D, further impacting cognitive functioning, self-care behaviors, and long-term metabolic control [20,21].

Finally, **non-use of diabetes management technologies**, such as continuous glucose monitors (CGMs) and insulin pumps, may adversely affect cognitive outcomes in children, primarily through poorer glycaemic control and increased daily disease burden. Managing diabetes without these devices may heighten stress for both children and caregivers, disrupt sleep, increase anxiety, and reduce treatment adherence, thereby indirectly impairing cognitive performance and academic functioning. However, it should be noted that device use may also negatively affect sleep due to alarm-related disturbances, particularly among caregivers [23,41].

#### 2.4.2. Protective Factors

Although a range of biological and psychosocial risk factors have been associated with adverse neurodevelopmental outcomes in paediatric diabetes, accumulating evidence suggests that several modifiable factors may confer resilience and mitigate potential brain-related sequelae.

Among all protective influences, **optimal and stable glycaemic control** appears most consistently associated with more favourable neurocognitive and neuroimaging outcomes. Lower HbA_1_c levels, greater time in range (TIR), and reduced exposure to both severe hypoglycaemia and chronic hyperglycaemia are linked to more typical trajectories of brain growth and cognitive performance. Longitudinal data from the DirecNet trial indicate that cumulative hyperglycaemic exposure correlates with reduced grey and white matter growth, suggesting that minimizing chronic dysglycaemia may help preserve normative developmental patterns [12]. Importantly, emerging interventional evidence demonstrates that improvements in TIR over relatively short periods may be accompanied by measurable changes in cognitive function and brain activation patterns, supporting the concept that at least part of diabetes-related brain vulnerability may be modifiable. Early optimisation of metabolic control—particularly soon after diagnosis and during periods of rapid brain maturation—may therefore have disproportionate protective benefit [42].

Use of **advanced diabetes technologies** like continuous glucose monitoring (CGM), insulin pump therapy, and hybrid closed-loop systems may indirectly protect brain health by reducing glucose variability and limiting extreme glycaemic excursions. By increasing TIR and decreasing the frequency and duration of hypoglycaemic and hyperglycaemic episodes, these technologies contribute to a more stable cerebral metabolic environment [29]. Beyond physiological stabilisation, technology use may reduce daily disease burden, treatment-related anxiety, and cognitive load associated with diabetes self-management. However, benefits may depend on consistent use, adequate education, and minimisation of sleep disruption caused by alarm fatigue. Thus, structured education and psychosocial support accompanying technology initiation are important components of its protective potential.

**Favourable psychosocial and family environment**, supportive, cohesive family context represents a major protective factor. Parental engagement in diabetes management, constructive communication, and low levels of family conflict are associated with better adherence, improved glycaemic control, and reduced psychological distress in children. Conversely, high parental anxiety, depression, or burnout may negatively influence metabolic stability and indirectly affect cognitive outcomes [18,19,20,21]. Family resilience may buffer the child from stress-related neuroendocrine activation (e.g., hypothalamic–pituitary–adrenal axis dysregulation) and promote consistent routines around glucose monitoring, insulin administration, sleep, and school engagement. Socioeconomic stability, parental education, and access to healthcare resources further strengthen this protective framework [43].

**Adequate sleep and circadian regulation** play a central role in memory consolidation, synaptic plasticity, emotional regulation, and metabolic homeostasis. Good sleep quality and regular sleep–wake cycles may attenuate neurocognitive vulnerability by stabilising glycaemic patterns and reducing stress hormone exposure. Minimising nocturnal hypoglycaemia and hyperglycaemia, optimising alarm settings, and promoting sleep hygiene practices are therefore clinically relevant not only for metabolic control but also for brain health preservation [44].

**Enriched cognitive environments**—including active school engagement, intellectual stimulation, and early identification of learning difficulties—may enhance neural plasticity and compensatory capacity. Even when subtle neurocognitive differences are present, timely neuropsychological evaluation and educational accommodations can help prevent secondary academic underachievement. Cognitive reserve, built through ongoing intellectual and social engagement, may mitigate the functional expression of mild structural alterations [45].

**Early diagnosis and prevention of DKA**, particularly at disease onset, is another potentially modifiable protective factor. Public awareness campaigns, early recognition of symptoms, and rapid access to paediatric care may prevent acute metabolic decompensation and its associated cerebral effects. Avoidance of recurrent DKA episodes during childhood further reduces cumulative neuroinflammatory and osmotic stress on the developing brain [35,37].

Finally, **integrated, structured multidisciplinary diabetes care**—including paediatric endocrinology, diabetes education, psychology, and when indicated neuropsychology—may provide a comprehensive protective framework. Routine monitoring of metabolic metrics, attention to mental health, and individualized support for high-risk subgroups (e.g., early-onset T1D, history of severe hypoglycaemia or presentation of DKA, persistent poor control) may reduce long-term neurocognitive burden [46,47].

Overall, protective factors in childhood diabetes extend beyond glycaemic targets alone. Brain health appears to be shaped by the interaction of metabolic stability, technological support, psychosocial resilience, adequate sleep, cognitive enrichment, and timely clinical intervention. Importantly, many of these influences are modifiable, underscoring the potential for preventive strategies aimed at preserving neurodevelopmental trajectories in children and adolescents with diabetes. Table 2 summarises the risk and protective factors associated with brain health in childhood type 1 diabetes.

### 2.5. Putative Pathophysiological Mechanisms

*Age at diabetes onset*. The vulnerability of the developing and still immature central nervous system may play a critical role in the emergence of later sequelae. During childhood, cerebral glucose utilisation and energy demand, as well as myelination processes, are markedly higher than in adulthood. Consequently, brain structures and functions may exhibit heightened sensitivity to early-life insults and to the metabolic disturbances present at diabetes onset [48]. As a result, structural and functional brain alterations and cognitive dysfunction may develop early, even before school age, particularly between 5 and 7 years of age [49].

*Chronic hyperglycaemia*. Sustained hyperglycaemia leads to the accumulation of advanced glycation end products, activation of the polyol pathway, and increased oxidative stress. These processes contribute to cell membrane damage, alterations in sphingolipid composition (including ceramides and sphingomyelin), disruption of cellular signalling pathways, and ultimately cell death [50,51,52,53,54].

*Hypoglycaemia*. Low blood glucose levels impair cerebral perfusion, particularly in frontal and hippocampal regions, resulting in disturbances in cognitive functioning. Severe hypoglycaemia induces upregulation of glucose transporters (GLUT1 and GLUT3), leading to excessive release of excitatory amino acids such as glutamate and aspartate, which in turn causes neuronal injury [55]. Recurrent hypoglycaemic episodes may result in cortical necrosis and, in regions such as the limbic system and hippocampus, increased glial cell proliferation [56].

*Blood glucose variability*. Glycaemic variability plays a significant role in the development of vascular and neural complications, including those affecting the central nervous system. Fluctuations in glucose levels lead to dysregulation of key intracellular signalling pathways, such as protein kinase C, protein kinase B, nuclear factor-κB, and mitogen-activated protein kinase [53,57]. Increased glycaemic variability and the associated disturbances in glucose transport contribute to neuronal damage. Oxidative stress appears to be a central mediator of glucose-induced neurotoxicity, leading to mitochondrial dysfunction and cell death [58,59].

*Ketoacidosis*. Diabetic ketoacidosis has both acute and long-term consequences. Cerebral oedema, impaired cerebral perfusion, elevated ketone and lactate concentrations, and reduced N-acetyl-L-aspartate levels have been associated with adverse long-term outcomes, with cytotoxic, vasogenic, and osmotic mechanisms all contributing [60,61,62]. Animal studies indicate that ketoacidosis induces chronic neuroinflammation, reactive gliosis, and microglial activation, thereby promoting the development of cognitive dysfunction and learning impairments over time [63,64].

*Insulin deficiency, cerebral metabolism, and insulin signalling*. Insulin exerts important neurotrophic effects, and insulin deficiency or fluctuating insulin levels may adversely affect cerebral metabolism and neural plasticity. In diabetes, brain glucose uptake, insulin transport, and cerebral energy metabolism may be altered. Emerging evidence suggests that disrupted insulin signalling and glucocorticoid exposure represent additional pathways contributing to diabetes-related brain injury [65].

**Figure 1:**
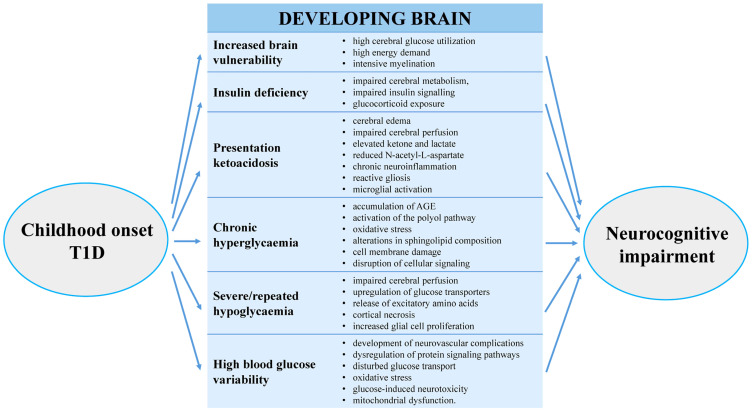
Pathophysiological mechanisms contributing to the brain dysfunction in children with type 1 diabetes [48,49,50,51,52,53,54,55,56,57,58,59,60,61,62,63,64,65,66].

In summary, multiple interacting metabolic, vascular, and inflammatory pathways likely underlie brain alterations in paediatric diabetes. Figure 1 depicts the putative pathophysiological mechanisms underlying brain damage in children with type 1 diabetes.

## 3. Type 2 Diabetes and the Brain

Type 2 diabetes is increasingly diagnosed in children and adolescents, raising significant concerns about its effects on the developing brain [66]. Unlike adults, young individuals with T2D experience metabolic dysfunction during critical periods of neurodevelopment characterized by rapid brain growth, synaptic pruning, and myelination. Growing evidence indicates that early-onset T2D may adversely affect brain structure, cognitive function, and mental health, with potentially enduring consequences.

*Hyperglycemia and neurodevelopment.* Chronic hyperglycaemia is a defining feature of T2D and has been implicated in neural injury through mechanisms including oxidative stress, mitochondrial dysfunction, and neuroinflammation. In the developing brain, sustained exposure to elevated glucose levels may disrupt normal neuronal maturation and synaptic plasticity. Evidence suggests that hyperglycaemia may disproportionately affect the hippocampus and prefrontal cortex—regions critical for memory, learning, and executive function [67].

*Insulin resistance and brain function.* Insulin acts as an important neuromodulator, contributing to neuronal survival, synaptic regulation, and neurotransmitter activity. Insulin resistance, a hallmark of T2D, may impair insulin signaling within the brain, resulting in reduced cognitive efficiency. In children and adolescents, disrupted central insulin action has been associated with deficits in attention, executive function, and processing speed, which may interfere with academic performance and daily functioning [68].

*Structural and functional brain changes* Neuroimaging studies in adolescents with T2D have demonstrated alterations in both gray and white matter. Findings of reduced gray matter volume and compromised white matter integrity suggest delayed or atypical brain maturation. Functional imaging studies have further identified altered connectivity within neural networks involved in self-regulation and cognitive control. Collectively, these results indicate that T2D may influence both brain structure and functional organization during development [69].

*Cognitive outcomes.* Youth with T2D often perform more poorly on neuropsychological assessments of memory, executive function, attention, and psychomotor speed compared with metabolically healthy peers. These cognitive domains are essential for learning, decision-making, and emotional regulation. Comorbid conditions such as obesity, hypertension, and sleep disturbances are common in paediatric T2D and may further exacerbate cognitive vulnerability [70].

*Mental health and emotional well-being.* Children and adolescents with T2D exhibit higher rates of depression, anxiety, and diabetes-related distress. Psychological stress can negatively influence brain development through dysregulation of the hypothalamic–pituitary–adrenal axis and increased inflammatory signaling. Poor mental health may also impair glycaemic control, creating a bidirectional relationship between emotional well-being and brain health [71].

*Inflammation and neurovascular effects.* Low-grade systemic inflammation associated with T2D may extend to the central nervous system. Proinflammatory cytokines can cross the blood–brain barrier, contributing to neuronal dysfunction and impaired cerebral perfusion. Early neurovascular alterations may increase the risk of subtle brain injury and predispose youth with T2D to long-term cognitive decline [72,73,74].

*Long-term implications.* Early exposure to metabolic dysfunction may increase vulnerability to accelerated cognitive aging and neurodegenerative disease later in life. Because neurodevelopmental disruptions during childhood and adolescence can have lasting effects, early diagnosis and optimal metabolic control are essential for preserving long-term brain health. Overall, T2D in children and adolescents poses a substantial risk to brain health through mechanisms involving hyperglycaemia, insulin resistance, inflammation, and psychological stress. Current evidence suggests that early-onset T2D may alter brain structure, impair cognitive function, and negatively affect mental health during critical stages of neurodevelopment. Early intervention strategies focusing on glycaemic control, mental health support, and lifestyle modification are therefore crucial to mitigating the neurological impact of T2D in youth.

In summary, emerging evidence indicates that paediatric type 2 diabetes is associated with both structural and functional brain alterations, underscoring a heightened vulnerability to early neurocognitive impairment.

## 4. Gaps in Knowledge and Future Directions

Despite the rapid expansion of research examining the relationship between childhood diabetes and brain health, substantial gaps remain that limit definitive conclusions and clinical translation.

*Causality, developmental trajectories, and long-term outcomes.* One of the most important unresolved issues concerns causality. Although longitudinal studies suggest that dysglycaemia is associated with altered trajectories of brain growth and cognitive performance, most available data are derived from cross-sectional analyses or prospective cohorts with relatively short follow-up periods. It remains unclear whether observed neuroanatomical and cognitive differences are a direct consequence of metabolic disturbances, whether they reflect pre-existing vulnerabilities related to genetic or familial factors, or whether they emerge through complex bidirectional interactions between metabolic control and neurodevelopment. Moreover, the stability and long-term significance of early structural and functional alterations are not yet fully understood. Whether early deviations in brain growth represent transient adaptations, delayed maturation, or progressive injury requires extended follow-up into adulthood. In particular, determining whether sustained improvement in glycaemic control can normalize or at least attenuate altered neurodevelopmental trajectories remains a critical unanswered question.

*Heterogeneity, moderators, and vulnerable subgroups.* Another major challenge lies in the substantial heterogeneity observed among children with diabetes. Effect sizes reported in neuroimaging and cognitive studies are typically small to moderate, suggesting that clinically meaningful impairment affects only a subset of individuals. However, reliable predictors of vulnerability and resilience are not yet well defined. Age at onset, particularly diagnosis during early childhood, may confer increased risk, yet recent findings have questioned the consistency of this association. Pubertal status, sex differences, socioeconomic background, sleep quality, psychosocial stress, parental mental health, comorbid conditions such as obesity or hypertension and early neurovascular complications likely interact in complex ways to influence neurodevelopmental outcomes. The interplay between biological susceptibility and environmental exposures remains insufficiently characterized. Large, harmonized, multicentre studies that integrate metabolic, neuroimaging, neuropsychological, genetic, and psychosocial data will be required to disentangle these interacting factors and to move toward more individualized risk stratification.

*Mechanistic biomarkers and biological pathways.* Although several pathophysiological mechanisms have been proposed, including oxidative stress, neuroinflammation, altered insulin signalling, mitochondrial dysfunction, and microvascular impairment, direct evidence in paediatric populations remains limited. The relative contribution of chronic hyperglycaemia, glycaemic variability, severe hypoglycaemia, and diabetic ketoacidosis to structural and functional brain changes has not been definitively quantified. Furthermore, it is not yet clear whether inflammatory and metabolic disturbances produce transient functional alterations or lead to persistent structural remodeling, particularly in white matter tracts undergoing active myelination. Advanced neuroimaging techniques, including diffusion-based metrics of microstructural integrity, perfusion imaging, and magnetic resonance spectroscopy, combined with circulating and cerebrospinal biomarkers of inflammation, oxidative stress, and blood–brain barrier function, may provide greater mechanistic insight. Translational integration between animal models and paediatric clinical cohorts will be essential to validate mechanistic hypotheses and identify modifiable biological pathways.

*Interventional and technology-driven studies.* Interventional evidence remains particularly scarce. While observational data consistently associate improved glycaemic stability with more favourable cognitive and neuroimaging outcomes, definitive proof that optimizing metabolic control alters brain developmental trajectories is lacking. Randomized and well-controlled studies examining early implementation of advanced diabetes technologies, such as hybrid closed-loop systems, with neurocognitive and neuroimaging outcomes as predefined endpoints are needed. Importantly, interventions initiated soon after diagnosis may be especially relevant, given the heightened neuroplasticity of the developing brain. Beyond glycaemic optimization, research should also evaluate adjunctive approaches targeting inflammation, insulin resistance, sleep disturbances, and psychosocial stress, as these factors may independently or synergistically influence brain health.

*Type 2 diabetes in youth: an urgent research priority.* The evidence base for type 2 diabetes in youth is even more limited than for type 1 diabetes. Paediatric T2D often occurs in the context of obesity, systemic inflammation, hypertension, sleep-disordered breathing, and socioeconomic disadvantage, all of which are independently associated with altered neurodevelopment. Disentangling the relative contributions of hyperglycaemia, insulin resistance, adiposity, and psychosocial adversity represents a major methodological challenge. Moreover, it remains unknown whether early-onset T2D confers an increased risk of accelerated cognitive ageing or earlier neurodegenerative processes. Given the rising global prevalence of youth-onset T2D and its potentially aggressive metabolic course, comprehensive longitudinal studies in this population represent an urgent research priority.

*Standardization and methodological challenges.* Methodological variability across studies further complicates interpretation. Differences in neuropsychological assessment tools, imaging acquisition protocols, definitions of severe hypoglycaemia and diabetic ketoacidosis, and the use of various glycaemic metrics limit cross-study comparability. Standardization of cognitive batteries, imaging parameters, and metabolic outcome measures would enhance reproducibility and facilitate meta-analytic synthesis. Inclusion of appropriate control groups, such as non-diabetic siblings, and careful adjustment for socioeconomic and familial confounders are also critical for reducing bias. Greater use of pre-registered protocols and data-sharing consortia would strengthen the methodological rigor of the field.

*Clinical translation and implementation science.* From a clinical translation perspective, there is currently no consensus regarding systematic neurocognitive screening in paediatric diabetes care. Although accumulating evidence supports the concept of brain health as a potential diabetes-related complication, practical guidance is lacking concerning which patients should be screened, at what intervals, and with which instruments. Implementation research is needed to determine how neurocognitive monitoring can be incorporated into routine care without imposing excessive burden on families or healthcare systems. Clarifying referral pathways to neuropsychology and educational support services will also be essential, particularly for high-risk subgroups.

*Lifespan perspective and prevention of later neurodegeneration.* Finally, a lifespan perspective is needed to determine whether subtle neurodevelopmental alterations in childhood diabetes predispose individuals to earlier cognitive decline in midlife or later-life neurodegeneration. Long-term follow-up extending beyond adolescence is required to establish whether early metabolic exposures have enduring consequences for brain ageing. Understanding whether optimal glycaemic control during childhood modifies lifelong cognitive trajectories will have profound implications for preventive strategies and health policy.

*Overall future direction.* In summary, the field must now move beyond documenting associations toward elucidating mechanisms, identifying modifiable risk pathways, and rigorously testing early interventions. Only through longitudinal, mechanistic, and translational research integrating metabolic, neurobiological, and psychosocial dimensions will it be possible to determine the extent to which diabetes-related alterations in the developing brain are preventable, reversible, or progressive.

## 5. Clinical Implications

Clinicians managing children with diabetes need to recognise that brain health is a potential target of diabetic complications. It seems to be important to monitor glycaemic control not only for classic complications but also for possible cognitive/brain consequences. Although cognitive dysfunction in children with diabetes appears clinically mild, it can significantly affect the quality of treatment. Neurocognitive deficits affect multiple cognitive domains, including executive function and processing speed. Evidence suggests that subtle brain injury may contribute to adverse psychological and mental health outcomes [75]. Impaired executive function and mental health can reduce treatment adherence and the capacity for adaptive lifestyle decision-making. These impairments may form a feedback loop in which diabetic dysglycemia leads to brain injury, further executive dysfunction, and worsening mental health, resulting in suboptimal adherence and continued dysglycemia [55]. Clinicians managing patients with poor glycaemic control should be aware of these interrelated factors.

Neurocognitive screening (attention, memory, executive function) especially in children with early onset, poor control, DKA history or frequent hypoglycaemia could be relevant during paediatric diabetes care. Promoting use of technologies (CGM, insulin pump) and glycaemic stability, reducing glucose variability and extremes should be an integral part of diabetes management. A supportive family environment, sleep hygiene, and cognitive stimulation especially in high-risk children could be relevant regarding prevention of neurocognitive dysfunction. Collaboration with neuropsychology/educational services if cognitive delays or attention deficits emerge is imperative.

## 6. Conclusions

The evidence, though still emerging, indicates that childhood T1D is associated with subtle but meaningful alterations in brain development, structure, and function, with corresponding cognitive implications. Hyperglycaemia, glycaemic variability, hypoglycaemia, younger age at onset, and DKA are among the risk factors. On the flip side, improved glycaemic stability and supportive psychosocial contexts offer protective potential. Future work must focus on longitudinal, mechanistic, and interventional studies to move from association to causation, and ultimately to mitigate brain-related sequelae in children with diabetes.

Type 2 diabetes in children and adolescents poses a significant risk to brain health through mechanisms involving hyperglycaemia, insulin resistance, inflammation, and psychological stress. Evidence indicates that early-onset T2D may alter brain structure, impair cognitive function, and negatively affect mental health during critical stages of neurodevelopment. Early intervention strategies targeting glycaemic control, mental health support, and lifestyle modification are crucial to mitigating the neurological impact of T2D in youth.

## Figures and Tables

**Table 1:** Cognitive domains and typical findings in paediatric type 1 diabetes.

Cognitive Domain	Typical Findings in Children with T1D vs. Controls	Notes on Risk/Modifiers
Full-scale IQ	Mildly lower	Effect size varies; influenced by control and duration [5]
Attention	Lower sustained attention, slower reaction time	Linked to glycaemic extremes and DKA [5]
Memory (verbal/visual)	Lower scores in some studies	Particularly with early onset and hyperglycaemia [14]
Executive function	Impairment in flexibility, inhibition, working memory	Emerging findings; imaging correlates present [23]
Psychomotor speed/motor	Slower responses, some fine motor deficits	Effects small but consistent [19]

**Table 2:** Summary of risk and protective factors for brain health in childhood T1D.

Risk Factors	Protective Factors
Chronic hyperglycaemia	Better glycaemic control
Severe hypoglycaemia episodes	Use of advanced diabetes technologies
High glucose variability	Family/caregiver support
DKA at diagnosis	Good sleep quality
Younger age at onset	Enriched cognitive environments
Non-use of diabetes management technologies	Early diagnosis and prevention of DKA
Psychosocial issues in parents/caregivers	Integrated multidisciplinary diabetes care
Poor sleep quality	

## Data Availability

No new data were created or analyzed in this study. Data sharing is not applicable to this article.
